# Using MODIS Land Surface Temperatures for Permafrost Thermal Modeling in Beiluhe Basin on the Qinghai-Tibet Plateau

**DOI:** 10.3390/s19194200

**Published:** 2019-09-27

**Authors:** Anyuan Li, Caichu Xia, Chunyan Bao, Guoan Yin

**Affiliations:** 1 College of Civil Engineering, Shaoxing University, Shaoxing 31200, China; tjxiaccb@126.com (C.X.); fengniaobcy@163.com (C.B.); 2 Key Laboratory of Rock Mechanics and Geohazards of Zhejiang Province, Shaoxing University, Shaoxing 31200, China; 3 State Key Laboratory of Frozen Soil Engineering, Cold and Arid Regions Environmental and Engineering Research Institute, Chinese Academy of Sciences, Lanzhou 730000, China; yinguoan123@126.com; 4 Key Laboratory of Geotechnical and Underground Engineering of Ministry of Education, Tongji University, Shanghai 200092, China

**Keywords:** remote sensing, permafrost thermal modeling, surface temperature, Qinghai-Tibet Plateau

## Abstract

It is essential to monitor the ground temperature over large areas to understand and predict the effects of climate change on permafrost due to its rapid warming on the Qinghai-Tibet Plateau (QTP). Land surface temperature (LST) is an important parameter for the energy budget of permafrost environments. Moderate Resolution Imaging Spectroradiometer (MODIS) LST products are especially valuable for detecting permafrost thermal dynamics across the QTP. This study presents a comparison of MODIS-LST values with in situ near-surface air temperature (*T_a_*), and ground surface temperature (GST) obtained from 2014 to 2016 at five sites in Beiluhe basin, a representative permafrost region on the QTP. Furthermore, the performance of the thermal permafrost model forced by MODIS-LSTs was studied. Averaged LSTs are found to strongly correlated with *T_a_* and GST with *R*^2^ values being around 0.9. There is a significant warm bias (4.43–4.67 °C) between averaged LST and T_a_, and a slight warm bias (0.67–2.66 °C) between averaged LST and GST. This study indicates that averaged MODIS-LST is supposed to be a useful data source for permafrost monitoring. The modeled ground temperatures and active-layer thickness have a good agreement with the measurements, with a difference of less than 1.0 °C and 0.4 m, respectively.

## 1. Introduction

Permafrost, which is defined as ground that remains frozen for two or more consecutive years, plays a crucial role in the energy and water cycle in cold regions [1,2,3]. Permafrost is thawing at the global scale in response to climate change [4] and, in turn, modify local and regional hydrological and ecological systems [3]. This trend will likely continue over the coming decades under the warming climate [5,6]. To assess its vulnerability related to climate change, it is essential to continuously map and monitor the thermal state of permafrost in the Qinghai-Tibet Plateau (QTP) [7]. However, it is not practical to monitor the permafrost thermal state using boreholes alone over the vast and remote QTP. In permafrost regions, the land surface temperature (LST) which measured from the satellites, is an important parameter for the energy budget of permafrost environments. To date, however, predicting the ground surface temperature (GST) remains a challenge in permafrost modeling because of the typical approach of employing the simplistic n-factor [3]. Here, GST is the in situ temperature at the surface of the ground, which is usually measured from the temperature sensors. Remote sensing techniques for permafrost monitoring tools are an ongoing development [8,9,10]. LST products derived from the Moderate Resolution Imaging Spectroradiometer (MODIS) provide daily radiometric LST values over large scale in swath and grid format. MODIS-LST products have been compared or validated against field temperature observations and increasingly being used to study land processes. Recent studies have used the satellite land surface temperature to monitor the temperature variability and warming trend in Arctic regions [11,12,13,14]. The application of MODIS-LST products highlights the prospects for large-scale permafrost monitoring at a high spatial resolution of 1 km on the QTP. However, permafrost is subsurface phenomenon that cannot be directly observed by remote sensing tools. The ground can remain unfrozen even if the temperature is negative. MODIS-LSTs are widely used to force the equilibrium or statistical permafrost model in the local and regional scale [15,16,17,18]. The majority of these studies can only provide a qualitative simulation of permafrost thermal state during a long-term period and cannot detect the thermal regime changes due to the surface temperature variations in a short period (e.g., <2 years). Marchenko et al. proposed that MODIS-LST could use as forcing datasets for a transient permafrost model [18]. Langer et al. tested the uncertainties of such LST-based permafrost modeling in a tundra lowland landscape on Samoylov Island [19]. For the permafrost terrain on the QTP, satellite-based LSTs are only used in equilibrium models [16,20]. However, the performance of such LSTs in transient permafrost models is unclear in different alpine ecosystems on the QTP.

It is necessary to assess the accuracy and precision of the LST products before we use them as forcing data. MODIS-LST validation is required to identify possible uncertainties in permafrost thermal monitoring and modeling on the QTP. Therefore, the primary goals of this study are: (I) to validate the MODIS-LST product in the permafrost environment on the QTP, (II) to test the performance of MODIS-LST for permafrost thermal modeling on the QTP. The propose of our work is to improve the understanding of uncertainties of the data in permafrost modeling, not to derive more accurate LSTs. 

## 2. Study Area

**Figure 1:**
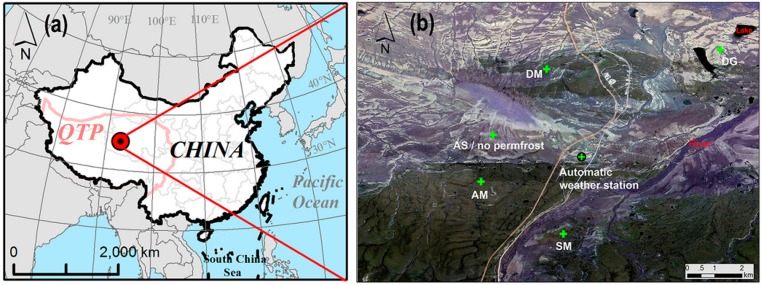
Location of the study area (**a**) and field sites on the QTP (**b**).

The research area locates in the Beiluhe basin (34.8° N, 92.9° E, 4628 m above sea level (a.s.l)), is a typical alpine ecosystem landscape with permafrost on the QTP (Figure 1a). The mean annual air temperature (MAAT) is −3.4 °C, and the total yearly precipitation is about 370 mm. It is snow-free during the whole year because rainfall mainly occurs between May and September [21]. The ground surface covered by a mixture of vegetation patches, exposed sand with gravel, and shallow water bodies. The regional permafrost features mean annual ground temperature around −1.5–0 °C, with an active layer thickness of 1.4–3.4 m. A large number of field measurements and long-term monitoring programs have created an available data basis on ground temperatures and climatological variables [21].

## 3. Method

### 3.1. In Situ Measurements 

The target area of in situ measurements is about 100 km^2^ (Figure 1b). One automatic climate monitoring station established by the State Key Laboratory of Frozen Soils Engineering between December 2001 and May 2002. The near-surface air temperatures (*T_a_*) at 2.0 m above the ground surface were measured using sensor HMP45C_L11 (Vaisala, Finland) in a solar radiation shield. Additionally, the station measured other meteorological variables such as precipitation, wind speed, and solar radiation. The station automatically recorded the data every 30 minutes using a data logger CR3000 (Campbell Scientific, Utah, USA). In the target area, the climate was assumed to be uniform in this study due to the relatively flat landscape [22]. 

In situ measurements of ground temperature performed in five representative ecosystem types [21]: swamp meadow (SM), alpine meadow (AM), degrading alpine meadow (DM), alpine steppe (AS), and desert grassland (DG). SM features wetland with shallow bodies. The vegetation is dominated by *Koeleria tibetica* with an average height of 8−15 cm. For AM, the vegetation cover is more than 0.7 and is dominated by *Kobrecia parva*. At DM, vegetation cover is between 0.3 and 0.8. AS is characterized by sand and gravels and normally features no permafrost. In DG, the surface is mainly covered by sand. Ground temperatures are measured in a 15 m borehole by the thermistors, which space at 0.1 m intervals in the upper 1.0 m, 0.5 m between 1.0 and 5.0 m depth, and 1.0 m below 5.0 m depth [21]. A CR3000 automatic data logger was used to record the measurements every six hours (0:00, 6:00, 12:00, 18:00 local time) at each site. Daily mean temperatures calculated from the recorded data for each site. The in situ measurements of ground temperature are available from 15 August 2014 to 14 August 2016. The area (1 km × l km) around the target site consists of approximately one homogeneous land cover type. Soil thermal properties (i.e., thermal conductivity) were measured using a portable thermal characteristic analyzer (KD 2 Pro, Decagon, USA). 

### 3.2. MODIS Clear-Sky LST Data

The level 3 MODIS-LST Collection 6 products (MOD11A1/MYD11A1, Version 6) obtained by the satellites Terra and Aqua used in this study. They feature a spatial resolution of 1 km × 1 km and provide four values of day and night LST from both the Terra (MOD) and Aqua (MYD) satellites based on the day-night split-window algorithm using MODIS bands 31 and 32 [23]. The Terra overpass time is about 10:30 (local time) in its descending mode and 22:30 in its ascending mode. The Aqua overpass time is around 13:30 in its ascending mode and 01:30 in its descending mode [24].

In this study, to evaluate the performance of MODIS-LST for a permafrost monitoring site, we use daily LST (MOD11A1/MYD11A1) from the available satellite data and compare them with the field observations. MODIS pixels centered on or located close to the field sites are selected for temperature comparisons. We use the Google Earth Engine Code Editor dataset to obtain and process the MOD11A1/MYD11A1 (https://code.earthengine.google.com) based on GPS (Trimble, CA, USA) records of the five sites. 

Here, one should note that LST is different from GST. GST is defined as the in situ temperature at the surface of the ground. The surface is usually covered by different materials, such as vegetation, organic layer, and so on. LST describes the land surface temperature of the earth (i.e., ‘skin’ temperature), which is measured from the satellites.

### 3.3. Permafrost Thermal Modeling

#### 3.3.1. Model description 

This study uses a numerical transient heat transfer model GIPL2 [25], which is capable of representing the freezing and thawing cycle in soil. This model solves the 1-D heat transfer equation accounting for phase change of soil water (Equation (1)). Heat conduction is assumed as the only process of energy transfer in the model.

(1) $$\frac{\partial \left(z,T\right)}{\partial t}-\frac{\partial }{\partial z}\left(k\left(z,T\right)\frac{\partial T\left(Z,T\right)}{\partial Z}\right)=0$$

where *T(z, t)* is the soil temperature (°C), *k(z,t)* is the soil thermal conductivity. *z* and *t* are the soil depth and time, respectively. *H(z, T)* is the enthalpy (Equation (2)):

(2) $$H\left(z,T\right)=\int_{0}^{T}C\left(z,s\right)ds+L\Theta \left(z,T\right)$$

where *C(z,s)* is the volumetric soil heat capacity (W/m^3^/°C), *L* is the volumetric latent heat of fusion (334 kJ/kg), Θ (*z, T*) is the the soil volumetric unfrozen (Equation (3)):

(3) $$\Theta \left(z,T\right)=\eta \left(z\right)\{\begin{array}{c} 1,T\geq T_{*}\\ a{\left|T\right|}^{-b},\;T<T_{*} \end{array}$$

where *a* and *b* are coefficients of the unfrozen water curve, *η(x)* is the volumetric moisture content, and T_*_ is a freezing point depression.

#### 3.3.2. Model operation

The soil and ground profile domain generated with 139 finite elements ranging from 0 to 100 m, vertically. The size of the cells increases with depths with a minimum grid cell spacing of 0.1 cm for the shallow soils and maximum spacing of 10 m at the bottom. The soil profile includes sand, clay, and bedrock (Table 1). The soil thermal parameters for each layer are listed in Table 1. All parameters required in the model are tested by fitting the model results to the measured ground temperature based on the measured daily GST.

The upper boundary conditions determine on time series of land surface temperature LST, which obtain from available clear-sky conditions (Section 3.2). The data gaps occurring due to clouds are filled by linear interpolation to get continuous data. The lower boundary conditions are defined based on the geothermal gradient of 0.07 °C/m, as estimated from a deep borehole [26]. To evaluate a realistic initial temperature profile, the numerical model is spun up using a repeated 10-year LST from 2004 to 2014 with a soil temperature profile.

### 3.4. Validation Datasets

Under clear-sky conditions, each MODIS LST data series (MOD11A1, MYD11A1) and the average value of MOD11A1 and MYD11A1 (MOD/MYD) from the MODIS pixel corresponding to the site locations are used to compare with in situ GST. The performance of the model validates by comparing the simulated soil temperature to in situ ground temperatures. Three statistical parameters calculated for comparing LST with GST and T_a_ from the field site and meteorological stations in this study. The correlation coefficient (*R*^2^) is used as a measure of the temporal coherence (co-variation in time) between LST and temperature measurements (GST and *T_a_*). The mean difference (MD) (T_a_—LST or GST—LST) is used as a measure of the difference between the two sets of data. The standard deviation of the MD (SD) is used to verify the variability around the mean MD.

## 4. Results 

### 4.1. Comparison between MODIS LST and in Situ GST and Ta

Due to lasting cloudy conditions over the study areas, LSTs from Terra (MOD) and Aqua (MYD) satellites are not continuously during the period of 15 August 2014–14 August 2016. Cloudless days of Terra and Aqua account for approximately 32% and 42% at the target area, respectively. When both Terra and Aqua (MODIS-LST) became available, the percentage of clear-sky condition decreases to about 20% to 23% during the study period.

In general, the LSTs are in good agreement with both T_a_ and GST on an annual basis in five sites. Table 2 presents the results of the comparison between LST and Ta/GST. The *R*^2^ values are all very high (>0.8). LSTs of MOD/MYD incredibly correlated with AT and GST (*R*^2^ around 0.9). The MDs between T_a_ and MODIS-LST ranged from −3.93 °C to −4.76 °C, which suggests that T_a_ is not a good proxy for surface temperature. The MDs between GST and MODIS-LST are between −0.12 °C and −2.32 °C at five sites, which illustrates that LST is slightly warmer than GST. At AM and DM, there is a slight cold bias during summertime. For sites AM, AS, and DG, MDs between MYD-LSTs and GSTs are smaller than that between MOD-LSTs and GSTs. This indicts that MYD-LSTs have a better performance than MOD-LSTs. For all sites, the SD values are relatively small (around 5 °C).

**Figure 2:**
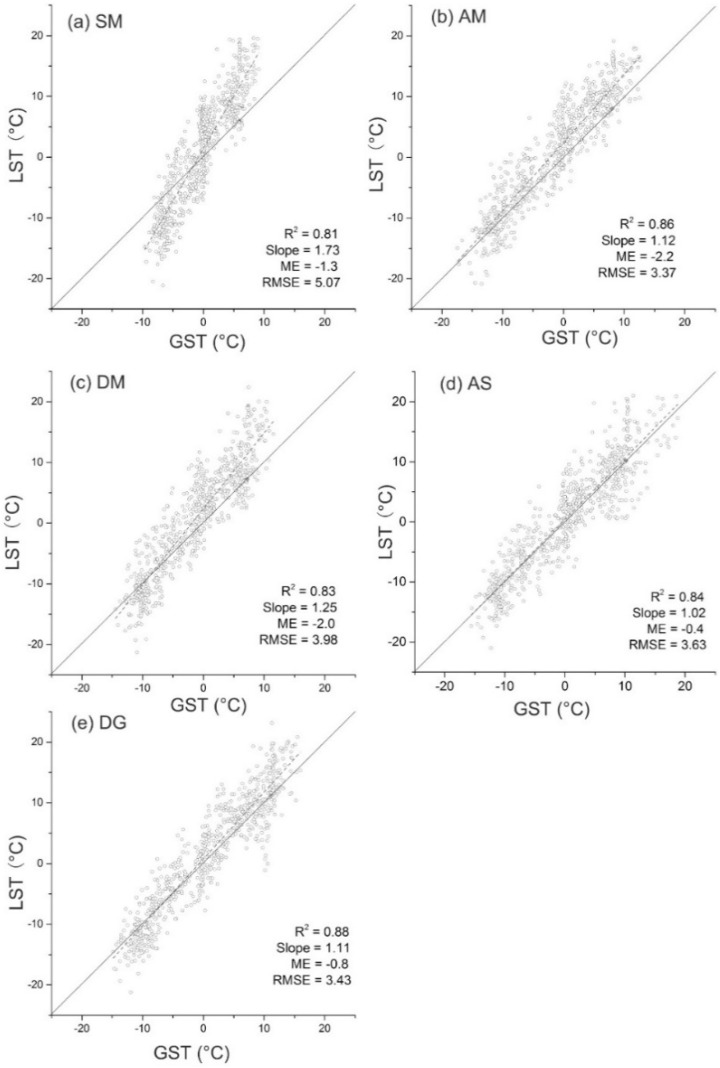
Comparison of daily LSTs with mean daily GST at site SM, AM, DM, AS, and DG during period of 15 August 2014–14 August 2016 (732 days). ME = mean errors.

To obtain continuous daily LST data, we use linear interpolation to fill the LST data gaps from available MODIS observations. The daily LST values are used to compare with the GSTs for five sites (Figure 2). There is a coherent relationship (R^2^ > 0.8) between the LSTs and field GSTs over the entire study period. The LSTs mainly distribute between −25 °C and 25 °C, while the GSTs show a spread in the range from −20 °C to 20 °C. On average, the daily LSTs are warmer than the GSTs with the mean errors (ME) ranging from −2.2 °C (site AM) to −0.8 °C (site DG). The data mostly center around the 1:1 line. However, for site SM, the LSTs are colder than the GSTs during wintertime (<0 °C), while warmer than that during summertime (>0 °C). This may attribute to the freezing and thawing of water in swamp meadow.

### 4.2. MODIS-LST-Based Modeling of Permafrost Temperature

**Figure 3:**
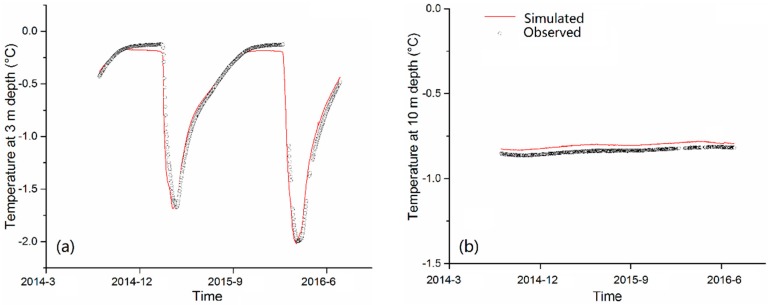
Comparison of the simulated and observed ground temperature at (**a**) 3.0 m depth, and (**b**) 10.0 m depth for site AM.

**Figure 4:**
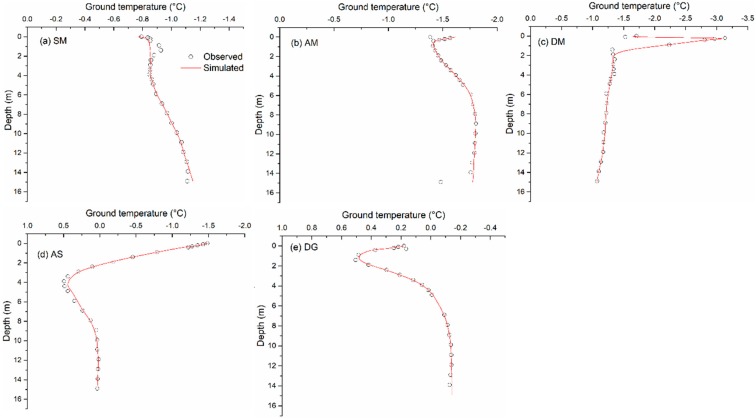
Comparison of the simulated and observed mean annual ground temperature profile for five sites: (**a**) site SM; (**b**) site AM; (**c**) DM; (**d**) AS; (**e**) DG.

Table 3 shows the model performance based on the MODIS-LST for soil depths of 3.0 m and 10 m. In general, the differences (ME) between simulated and observed ground temperature are below 1.0 °C. Most of the *R*^2^ values are above 0.8, with the mean errors (ME) ranging from 0.02 °C (Site DG at 10 m) to 0.82 °C (Site AS at 3 m). At a depth of 3.0 m, the general magnitude of temperature dynamics can be well reproduced. Compared to the in situ measurements, the modeled temperatures at 10 m depth are slightly warm, as shown in Figure 3 for site AM. Other comparisons not shown here. As shown in Figure 4, the annual mean ground temperature profile simulated by the thermal model have a good agreement with the measurements at the five sites. For sites SM and AM, there are apparent differences at shallow depths. Simulated and measured maximum thaw depths (i.e., ALT) based on the 0 °C isotherm are presented in Table 4. In most cases, the difference between measured ALT and simulated ALT is lower than 0.2 m. For site SM, the model overestimates the ALT by about 0.35 m.

## 5. Discussion

This study illustrates that the MODIS-LST has a good agreement with ground surface temperature (*R*^2^ > 0.87, mean MD = −1.5 °C) in different land surface type. However, existing cloud cover makes it not possible to measure LST from the satellites. Available LST values cover only around 20% of the study period by calculating the daily mean of both available Aqua and Terra values. Additionally, there are still some shortcomings of MODIS cloud detection algorithm, which have been pointed out over Arctic regions [27,28,29]. Further study should be conducted on the improvement of the cloud detection algorithm. Accurate temporal LST averages require for permafrost monitoring and modeling. In the present state, a reliable gap filling algorithm is highly desirable. Hachem et al. employ a possible gap filling procedure using a sinusoidal model to map the surface temperature and permafrost distribution [30]. Ran et al. propose a pragmatic scheme to estimate the mean annual surface temperature based on MODIS Aqua/Terra LST products [20]. Reanalysis products are also an approach to fill the gap when the MODIS-LST data is sparse. In this study, linear interpolation approach can fill the gaps and provides continuous data. 

The comparison of LST to *T_a_* indicates a warm bias of more than 4.0 °C in most field sites. This result illustrates that near-surface air temperature is not a good proxy for ground surface temperature on the QTP. In Arctic permafrost regions, some studies referring to MODIS validation demonstrated a general cold bias [29,31]. To the contrary, this study demonstrates a warm bias in different land surface type. At SW, AS and DG, there is a slight warm bias around 1.0 °C. At AM and DM, this bias is around 2.5 °C. The primary potential source of cold preferences in the Arctic is the presence of clouds, which may lead to a low temperature. In our study, LST presents clear-sky skin temperature. However, GST shows the temperature at 5 cm depth, where the impacts of weather and solar radiation can remove. Ground temperature in this shallow layer is expected to vary in time and space due to soil moisture [32]. Additionally, the differences between LST and GST (in situ LST) can be attributed to the fact that MODIS pixel may character different surface roughness over the area of 1 km × 1 km. Lin et al. have made density ground thermal measurements over a local-scale area (100 m^2^) in the different ecosystem on the QTP. He found land surface temperature variability of around 1.0 °C in each land cover type [22].

The average of all satellite observations (i.e., four times per day) is a good proxy for ground surface temperature for permafrost monitoring at a 1-km scale. Heterogeneity of the surface type and the soil moisture conditions cannot be resolved in 1 km^2^ by MODIS-LST. However, the warm bias (i.e., LST is warmer than GST) may constitute an important error source, when MODIS-LST is used to force the permafrost thermal model. Our assessment focuses on the performance of MODIS-LST products for two years. An evaluation of the performance of long-term LST products using gap filling approach will be of great importance on the remote QTP. 

The results of the permafrost model demonstrate that MODIS-LST can provide a useful upper boundary for thermal modeling of permafrost in different ecosystems on the QTP. There are relatively large errors between the simulated and measured ground temperatures at site SM in shallow depth. On the one hand, this may be the response to the uncertainty of the LST. On the other hand, this can be attributed to the impact of soil water dynamics and organic in the upper soil layer. This study tested the uncertainties in different ecosystems and soil types. In recent studies, ecotypes are usually used to scale up the soil characteristics for spatial permafrost thermal modeling at large scale [33,34,35]. This study is useful for landscapes on the QTP that feature similar subsurface when the MODIS-LST is used to drive the permafrost model. Therefore, the MODIS-LST products provide a valuable data source for permafrost modeling in ground temperature and active-layer thickness on the QTP. In addition, the impact of surface heterogeneities such as forest, snow cover, lakes on the MODIS LST and ground thermal regime is not accounted for. Further studies should be performed for different landscape types and permafrost model requires significant improvements in order to represent surface heterogeneities.

## 6. Conclusions

In this study, we compare the average LST acquired from different MODIS products to in situ daily mean GST and *T_a_* in diverse alpine ecosystems in Beiluhe basin, a permafrost region on the Qinghai-Tibet Plateau. A thermal permafrost model forced by the valid MODIS-LST is used to simulate the ground thermal state. From this study, the following conclusions can be drawn:

(1) MODIS-LST product is a beneficial data source for permafrost thermal monitoring on the Qinghai-Tibet Plateau at the regional scale. Mean daily LSTs have a stronger correlation with *T_a_* and GST when the two satellites Terra and Aqua data combined than that taken from one. 

(2) The averaged MODIS-LST have a slight warm bias (around 1.0 °C) at swamp meadow, alpine steppe meadow, and desert grassland, but a sizeable warm bias (approximately 2.5 °C) at alpine meadow ecosystem.

(3) The inter-annual variations in permafrost temperature and thaw depth can be simulated from valid MODIS-LST products in different alpine ecosystems on the QTP. The uncertainties of permafrost modeling based on MODIS-LST are less than 1.0 °C in temperatures at different soil depth, and less than 0.4 m in active-layer thickness. 

## Figures and Tables

**Table 1:** Ground properties for each soil layer given. VWC = volumetric water content (fraction of 1); UWC = unfrozen water coefficients; *C_t_/C_f_* = thawed/frozen volumetric heat capacities; *k_t_/k_f_* = thawed/frozen thermal conductivities.

Soil Layer	VWC	UWC		*C* (10^6^ Jm^−3^K^−1^)		*K* (Wm^−1^K^−1^)		Depth (m)
		a	b	*C_t_*	*C_f_*	*k_t_*	*k_f_*	
**SM**								
Sand	0.20	0.07	−0.17	3.1	1.5	1.5	0.9	0–1
Clay	0.18	0.12	−0.15	2.5	1.9	1.7	2.2	1–10
Rock	0.04	0.01	−0.1	3.25	2.48	2.7	3.1	>10
**AM**								
Sand	0.18	0.07	−0.17	3.1	1.5	1.6	2.4	0–2
Clay	0.17	0.12	−0.15	2.5	1.9	0.7	1.4	2–10
Bedrock	0.04	0.01	−0.1	3.25	2.48	2.7	3.1	>10
**DM**								
Sand	0.06	0.037	−0.14	2.8	2.2	1.3	1.6	0–2
Clay	0.12	0.12	−0.15	2.5	1.9	1.3	1.6	2–10
Rock	0.04	0.01	−0.1	3.25	2.48	2.7	3.1	>10
**AS**								
Sand with gravel	0.12	0.037	−0.14	2.8	2.2	1.3	1.6	0–3
Clay	0.12	0.12	−0.15	2.5	1.9	0.6	1.0	3–10
Rock	0.04	0.01	−0.1	3.25	2.48	2.7	3.1	>10
**DG**								
Sand	0.1	0.05	−0.17	3.1	1.5	1.3	1.6	0–5
Clay	0.12	0.12	−0.15	2.5	1.9	0.6	1.0	5–10
Rock	0.04	0.01	−0.1	3.25	2.48	2.7	3.1	>10

**Table 2:** Statistics of Correlation Coefficient (*R*^2^), Mean Difference (MD), Standard Deviation (SD) between LST and AT/GST for daily average from Aqua (MOD) and Terra (MYD) separately, and Aqua/Terra (MOD/MYD) combined.

Site		R^2^	MD (°C)	SD (°C)
		*T_a_*/GST	*T_a_*/GST	*T_a_*/GST
SM	MOD	0.87/0.81	−4.57/−0.98	3.61/5.65
	MYD	0.88/0.82	−4.38/−0.98	3.45/5.64
	MOD/MYD	0.94/0.87	−4.65/−0.81	2.61/5.39
AM	MOD	0.85/0.86	−4.43/−2.58	3.66/3.73
	MYD	0.87/0.86	−3.99/−2.32	3.46/3.71
	MOD/MYD	0.92/0.91	−4.43/−2.66	2.76/3.11
DM	MOD	0.85/0.83	−4.14/−2.21	3.80/4.46
	MYD	0.86/0.84	−4.48/−2.62	3.51/4.22
	MOD/MYD	0.91/0.89	−4.46/−2.53	2.89/3.95
AS	MOD	0.85/0.83	−4.58/−0.91	4.03/3.98
	MYD	0.85/0.85	−3.93/−0.12	3.88/3.75
	MOD/MYD	0.92/0.91	−4.67/−0.67	3.02/2.98
DG	MOD	0.86/0.88	−4.76/−1.22	3.99/3.65
	MYD	0.85/0.86	−4.47/−0.62	4.03/3.84
	MOD/MYD	0.92/0.93	−4.73/−1.05	3.12/2.88

**Table 3:** Statistics of the correlation coefficient (R^2^), mean errors (ME), and root mean square errors (RMSE) between simulated and measured ground temperature at depth of 3.0 m and 10.0 m for five sites.

Site	Depth	R^2^	ME	RMSE
SM	3	0.90	0.12	0.35
	10	0.93	0.11	0.25
AM	3	0.96	0.11	0.32
	10	0.92	0.10	0.26
DM	3	0.94	0.10	0.21
	10	0.75	0.05	0.02
AS	3	0.91	0.82	0.53
	10	0.89	0.08	0.22
DG	3	0.83	0.06	0.1
	10	0.90	0.02	0.1

**Table 4:** Differences between simulated ALT and measured ALT for the five sites.

Site	Simulated ALT (m)	Measured ALT (m)	Difference (m)
SM	1.75	1.40	0.35
AM	1.90	1.80	0.10
DM	1.60	1.80	0.20
AS	No permafrost	No permafrost	/
DG	3.20	3.40	0.20
